# Physiological evolution during adaptive radiation: A test of the island effect in *Anolis* lizards

**DOI:** 10.1111/evo.13741

**Published:** 2019-04-23

**Authors:** Jhan C. Salazar, María del Rosario Castañeda, Gustavo A. Londoño, Brooke L. Bodensteiner, Martha M. Muñoz

**Affiliations:** ^1^ Facultad de Ciencias Biológicas Departamento de Ciencias Naturales Universidad Icesi Cali Valle del Cauca Colombia; ^2^ Department of Biological Sciences Virginia Tech Blacksburg Virginia 24061; ^3^ Facultad de Ciencias Biológicas Departamento de Ciencias Naturales Universidad del Valle Cali Valle del Cauca Colombia

**Keywords:** Adaptive radiation, *Anolis*, Bogert effect, lizards, physiological evolution, thermal physiology

## Abstract

Phenotypic evolution is often exceptionally rapid on islands, resulting in numerous, ecologically diverse species. Although adaptive radiation proceeds along various phenotypic axes, the island effect of faster evolution has been mostly tested with regard to morphology. Here, we leveraged the physiological diversity and species richness of *Anolis* lizards to examine the evolutionary dynamics of three key traits: heat tolerance, body temperature, and cold tolerance. Contrary to expectation, we discovered slower heat tolerance evolution on islands. Additionally, island species evolve toward higher optimal body temperatures than mainland species. Higher optima and slower evolution in upper physiological limits are consistent with the Bogert effect, or evolutionary inertia due to thermoregulation. Correspondingly, body temperature is higher and more stable on islands than on the American mainland, despite similarity in thermal environments. Greater thermoregulation on islands may occur due to ecological release from competitors and predators compared to mainland environments. By reducing the costs of thermoregulation, ecological opportunity on islands may actually stymie, rather than hasten, physiological evolution. Our results emphasize that physiological diversity is an important axis of ecological differentiation in the adaptive radiation of anoles, and that behavior can impart distinct macroevolutionary footprints on physiological diversity on islands and continents.

Ever since Darwin's journeys to the Galápagos in the 19th century, islands have been widely recognized as important evolutionary cradles for biodiversity (Grant and Grant 2008; Losos and Ricklefs 2009). Islands are home to many strikingly diverse adaptive radiations such as the Hawaiian silverswords and honeycreepers, Caribbean *Anolis* lizards, and Darwin's finches from the Galápagos (Lack 1947; Carlquist 1974; Schluter 2000; Grant and Grant 2008; Losos 2009). The exceptional phenotypic diversity of many island radiations is thought to be triggered by “ecological opportunity”: as a result of fewer predators and competitors, diversification is predicted to be exceptionally rapid in island environments (Simpson 1953; Schluter 2000; Gillespie et al. 2001; Gavrilets and Losos 2009; Mahler et al. 2010). In contrast, phenotypic diversification in mainland radiations should be limited by community saturation and stronger predation pressures, resulting in slower evolution (Schluter 1988).

Despite strong conceptual support for the island effect of faster phenotypic evolution, results from empirical studies explicitly comparing patterns of island and mainland evolution are equivocal. Although some studies report faster evolution in island lineages (e.g., Lovette et al. 2002; Millien 2006; Ackerly 2009; Garcia‐Porta et al. 2016), others either find comparable patterns of evolution among landmasses or faster rates in mainland lineages (e.g., Bromham and Woolfit 2004; Arbogast et al. 2006; Pinto et al. 2008; Raia and Meiri 2011). Studies of the island effect, however, have been almost exclusively focused on comparisons of morphological characters, whereas adaptive radiation typically occurs along numerous phenotypic axes including morphology, physiology, and behavior (Schluter 1996, 2000; Givnish et al. 2004; Ackerly et al. 2006; Velasco et al. 2016). Thus, examining the evolutionary dynamics of physiology would greatly enrich our understanding of how phenotypic diversity arises on islands and continents.

*Anolis* lizards provide an ideal system to test the island effect in physiological evolution. This genus, comprising more than 400 species, is widely distributed on both islands (Caribbean and Pacific islands) and in mainland habitats (mainland North, Central, and South America) (Losos 2009; Poe et al. 2017). Although the evolutionary dynamics of adaptive radiation have been best studied with respect to morphology (Harmon et al. 2003; Pinto et al. 2008; Mahler et al. 2010; Caetano and Harmon 2018), anoles are also physiologically diverse (Velasco et al. 2016, 2018; Gunderson et al. 2018). In the Caribbean, different microhabitat specialists, termed as “ecomorphs,” evolved early in the radiation, resulting in assemblages of closely related, but morphologically distinct lizards. This was followed by physiological specialization within ecomorph clades to different thermal environments (Ruibal 1961; Rand 1964; Williams 1972; Losos 2009; Hertz et al. 2013). For example, species from the same ecomorph avoid competition in sympatry by partitioning the thermal niche (e.g., through different shade use), resulting in communities of closely related organisms with little overlap in physiological characteristics (Ruibal 1961; Rand 1964; Gunderson et al. 2018). In addition to thermal microhabitat partitioning, anoles are distributed across wide elevational gradients (from sea level to over 2000 m) on both islands and the mainland, which has resulted in physiological specialization to different thermal extremes (van Berkum 1986; Losos 2009; Muñoz et al. 2014). Importantly, the extent to which “ecomorphs” are limited to island habitats is unclear (e.g., Muñoz et al. 2015; Moreno‐Arias and Calderón‐Espinosa 2016), suggesting that mainland habitats may provide as much opportunity as islands for rapid evolution.

The role of thermoregulatory behavior in mediating patterns of physiological evolution on mainland and island habitats is also unexplored. Ectotherms, such as lizards, can behaviorally select thermal microhabitats to which they are already physiologically well adapted, thus reducing exposure to selection and limiting physiological differentiation across environments (discussed in Huey 1982; Stevenson 1985; Angilletta 2009). By buffering organisms from selection, behavioral thermoregulation can result in slower physiological evolution (a phenomenon also known as “behavioral inertia” or the “Bogert effect”; Huey et al. 2003). The Bogert effect is particularly apparent in upper physiological features of the thermal performance curve, such as heat tolerance, because thermoregulatory behavior can be especially effective when temperatures are heterogeneous (e.g., during the day). As a consequence, upper physiological limits tend to exhibit slower rates of evolution than lower physiological limits (e.g., Sunday et al. 2011; Bozinovic et al. 2014; Muñoz et al. 2014).

Thermoregulatory behavior varies extensively in reptiles, as it reflects the trade‐offs between its benefits (such as higher maximal performance) and its costs (such as time investment and exposure to predators), and these variables often change among habitats (Huey 1974; Huey and Slatkin 1976; Angilletta et al. 2002, 2009). Many aspects of anole behavior differ between mainland and island anoles (Irschick et al. 1997; Perry 1999; Losos 2009). For example, mainland anoles move around their environments substantially less frequently than their island counterparts (Perry 1999). If lower movement rates on the mainland are correlated with behavioral passivity (e.g., a stronger correlation between body temperature and local thermal environment), then physiological evolution might also differ between mainland and island anoles. Another possibility, however, is that rates of physiological evolution might reflect differences in climatic niche turnover. Previous work by Velasco et al. (2016) found that Caribbean anoles exhibited narrower climatic niche breadths and faster rates of overall diversification than mainland species. Correspondingly, physiological evolution might be expected to be more rapid on islands than on the mainland.

In this study, we assembled the largest *Anolis* physiology database to date (120 species) for three key physiological traits: cold tolerance (*CT_min_*), field‐active body temperature (*T_b_*), and heat tolerance (*CT_max_*). We addressed three central aims. First, we modeled the dynamics of physiological and climatic niche evolution in island and mainland lineages to empirically test for an island effect of faster divergence. Then, we considered how differences in thermoregulatory behavior between mainland and island environments might mediate the dynamics of physiological evolution. Consistent with the Bogert effect, we expected that more stable body temperatures across habitats should be associated with slower physiological evolution. Finally, we considered the interactions between behavior, local thermal environment, and physiology in the adaptive radiation of anoles and, more broadly, in shaping patterns of phenotypic diversification on both islands and continents.

## Materials and Methods

### STUDY SPECIES AND DATA COLLECTION

We gathered physiological data from previously published and unpublished work (Table S1). Species were included in our analysis if we could find data for at least one of the following physiological traits: cold tolerance (critical thermal minimum, *CT_min_*), field‐active body temperature (*T_b_*), and heat tolerance (critical thermal maximum, *CT_max_*). In ectotherms, such as lizards, the ability to perform a task (such as sprinting) is contingent on body temperature, such that performance is maximized over a range of temperatures and decreases at higher and lower temperatures until the animal is immobilized (Huey 1982; Angilletta 2009). *CT_min_* and *CT_max_* refer to the lower and upper thermal bounds of locomotor function, and are widely used metrics for physiological tolerance limits in ectotherms (Spellerberg 1972; Huey 1982; Lutterschmidt and Hutchison 1997; Angilletta 2009). For all species, *CT_min_* and *CT_max_* were experimentally estimated as the lower and upper temperatures, respectively, at which a lizard failed to right itself when flipped on its back (Spellerberg 1972). Physiological variables are often subject to considerable noise (e.g., Camacho and Rusch 2017). To minimize noise, we excluded any measures of thermal limits gathered through different experimental end points (e.g., the onset of muscle spasms or lethal limits; Lutterschmidt and Hutchison 1997). We gathered *CT_min_* and *CT_max_* values from previously published ectotherm databases (Huey et al. 2009; Sunday et al. 2011), and supplemented those data with additional searches of more recent work. Intraspecific variation across geographic clines is highly relevant when assessing physiological variation. Whenever data were available from multiple locations, we accounted for intraspecific variation by weighting the trait mean by sample size in each location, with greater weight given to localities with greater sampling.

Body temperature, *T_b_*, refers to the field‐measured core temperature of active lizards (i.e., individuals that are not hiding or sleeping). In diurnal lizards, such as anoles, *T_b_* correlates strongly with thermal habitat choice (behavior) and optimal sprinting temperature (performance), thus reflecting a species' intrinsic thermal sensitivity (Huey et al. 2012). We gathered anole *T_b_* values from a published database (Hertz et al. 2013), and added additional data from our own searches of recent work. Following Hertz et al. (2013), we only included data for body temperature measurements that were gathered during the normal (daytime) hours of activity for the species. We included data from all seasons, and from both males and females. We did not include data from juveniles. For Anolis *sagrei*, a species that is native to Cuba and the Bahamas (islands) and invasive in North America (mainland), we focused only on data collected from its ancestral range on islands. As with *CT_min_* and *CT_max_*, we accounted for intraspecific variation in *T_b_* by weighting the trait mean by sample size in each location, with greater weight given to localities with greater sampling.

Field‐active body temperature can vary due to a number of factors, such as time of day and weather conditions (Angilletta 2009; Vickers 2014). To reduce noise in this variable, we followed the approach of Hertz et al. (2013) by considering 10 minimum observations to provide robust support for body temperature. For 19 of the 101 of species in our *T_b_* database, there were fewer than 10 measurements of body temperature. As such, we conducted our evolutionary analyses (described next) twice for body temperature, once for the whole dataset and again for the subset of species for which the number of observations was ≥10.

### EXTRACTING BIOCLIMATIC DATA FOR MAINLAND AND ISLAND SPECIES

To test how environment may contribute to patterns of physiological evolution among landmasses, we compared thermal conditions between mainland and island habitats. For every georeferenced sampling locality in our physiological database, we extracted all temperature variables (bio 1–bio 11; Table S2) from the environmental layers available through the WorldClim database (Hijmans et al. 2005). These variables summarize thermal averages, extremes, and ranges, as well as seasonality trends. As with our averages of the physiological traits, we weighted our estimates of environmental variables by the number of individuals measured in each site. Thus, if a species was measured in multiple localities, thermal variables reflected the average variation among those sites, with greater weight given to localities with more trait measurements. We restricted this analysis to the 115 species (62 island and 53 mainland) for which we could reliably georeference capture locality.

### COMPARING PHYSIOLOGICAL AND CLIMATIC EVOLUTION BETWEEN MAINLAND AND ISLAND ANOLES

**Figure 1:**
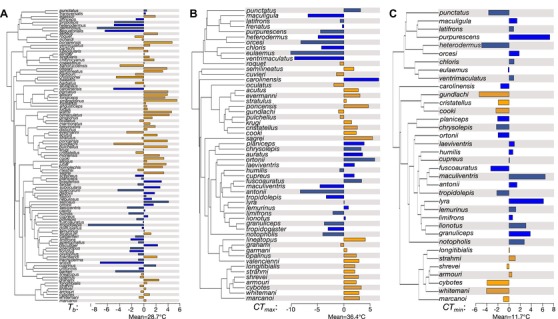
Phylogeny of *Anolis* lizards showing the relationships among the island (orange) and mainland (blue) species used in this study. Each panel depicts species values (relative to overall trait mean) for heat tolerance (*CT_max_*), body temperature (*T_b_*), and cold tolerance (*CT_min_*).

All analyses were performed in R (R Development Core Team 2014). We used the time‐calibrated tree of Poe et al. (2017), which we pruned from 379 species to the 120 species that were analyzed in this study (Fig. 1). This dataset consisted of 56 species from mainland habitats (North America, Central America, and South America) and 64 species from islands (Greater Antilles, Lesser Antilles, Great Bahama bank, Pacific islands). In brief, the tree was constructed from a Bayesian analysis of genetic data (50 loci representing 24,817 sites) and morphology (46 characters). The time calibration points used to create a chronogram were based on fossil data (Conrad et al. 2007; de Queiroz et al. 1998) using the relaxed‐clock approach, which allowed for rate variation among lineages.

To account for strong collinearity among climatic variables and relatedness among taxa, we reduced the dimensionality of the data using a phylogenetic principal component (PC) analysis, and used the lambda method to obtain the correlation matrix, which we implemented using the *phyl.pca* function in the phytools package (Revell 2012). We compared PC values between mainland and island taxa using ANOVA, with landmass as a fixed effect.

We tested whether landmass (“mainland” vs. “island”) was associated with different patterns of physiological (*CT_min_, T_b_*, and *CT_max_*) and climatic (PC 1, PC 2, and PC 3; see Results) evolution by fitting Brownian motion (BM) and Ornstein‐Uhlenbeck (OU) models. Collectively, these models contained three key parameters describing physiological evolution: the rate of stochastic trait evolution (σ^2^), the evolutionary trait optimum (θ), and the rate of adaptation to the optimal state (α). We first sampled potential histories for species’ landmass in proportion to their posterior probability (Huelsenbeck et al. 2003) by creating 500 stochastic character maps with the *make.simmap* function in phytools (Revell 2012), and then integrated each parameter estimate over the total sampled histories. Using the R package OUwie (Beaulieu et al. 2012), we then fitted a total of five different models. One or two evolutionary parameters (θ, σ^2^) were estimated for the clade as a whole, or separately for island and mainland species. The simplest model is a single‐rate BM in which a single σ^2^ was estimated for the whole clade. The multi‐rate Brownian Motion (BMS) model is also a BM model, but which estimated σ^2^ separately for mainland and island species. OU1 is an OU model that fitted a single trait optimum (θ) for the whole clade. The OUM model fitted separate θ for mainland and island anoles, while estimating a single σ^2^ for the entire clade. Finally, the OUMV model is an OU model that allowed both rate and optimum trait value parameters to vary between island and mainland species.

We fitted these five models separately for each physiological trait (*CT_min_*, *T_b_*, and *CT_max_*), each climatic habitat variable (PC 1, PC 2, and PC 3), and assessed support for model fit using sample size‐corrected AIC_C_. Any model(s) with ∆AIC_C_ ≤ 4 were considered to have equal support (Burnham and Anderson 2002).

For each of our physiological traits, we also calculated the phylogenetic half‐life (*t*_1/2_) of a single optimum OU1 model fitted separately for island and mainland taxa. *t*_1/2_ (estimated as log(2)/α) describes how much time is required for a lineage to get halfway to its phenotypic optimum, θ (Hansen et al. 2008; Münkemüller et al. 2015). A small *t*_1/2_ (relative to the length of the tree) indicates that phylogenetic information is either lost rapidly or that phenotypic values oscillate around a mean with a narrow variance, whereas half‐lives approaching or exceeding the length of the tree converge on a BM model of trait evolution. We also calculated stationary variance (*V_y_*), a dispersion parameter describing the equilibrium variance around the optimum (Hansen 1997). *V_y_* is calculated as σ^2^/(2α), where α describes the selective pull (rubber band parameter) to the trait optimum. Greater *V_y_* indicates greater variance in the optimal trait value, θ.

### BODY TEMPERATURE‐ENVIRONMENT RELATIONSHIPS BETWEEN LANDMASSES

To test whether the relationship between local thermal environment and lizard body temperature differed between mainland and island anoles, we used phylogenetic multiple regression analysis in which all climatic habitat variables (PC 1, PC 2, and PC 3; see Results) and landmass (island vs. mainland) were considered predictors of mean body temperature. Phylogenetic regression assumes that branch length is proportional to residual error in the model (Felsenstein 1985; Revell 2010). As such, we performed phylogenetic generalized least squares (PGLS) regression in which we simultaneously estimated phylogenetic signal, λ (Pagel 1999) in the residual error with the regression parameters (Revell 2010) using the *gls* function in the R package nlme (Pinheiro et al. 2018). We used the *stepAIC* function in the R package MASS (Ripley et al. 2013; Venables and Ripley 2013) to compare models via stepwise addition and removal of predictors.

## Results

### SUMMARY OF PHYSIOLOGICAL AND ENVIRONMENTAL VARIABLES

In total, we gathered physiological data from 120 species of *Anolis* lizards, 56 of which are mainland species and 64 of which are from islands (Fig. 1, Table S1). In our phylogenetic PC analysis of the WorldClim thermal variables, we recovered three PC axes with eigenvalues >1 that, together, explain 84% of the variation in the environmental data (Table S3). The first axis (PC 1) loaded strongly with average trends in thermal environment. These include mean annual temperature (MAT; loading = 0.979), maximum temperature of the warmest month (0.810), minimum temperature of the coldest month (0.965), mean temperature of the wettest quarter (0.912), and mean temperature of the driest quarter (0.977). The second axis (PC 2) loaded strongly with temperature seasonality (0.842) and annual temperature range (0.860). The third axis (PC 3) loaded highly with mean temperature of the warmest quarter (−0.966), and mean temperature of coldest quarter (−0.927).

### TESTING FOR THE ISLAND EFFECT IN PHYSIOLOGICAL AND CLIMATIC EVOLUTION

**Figure 2:**
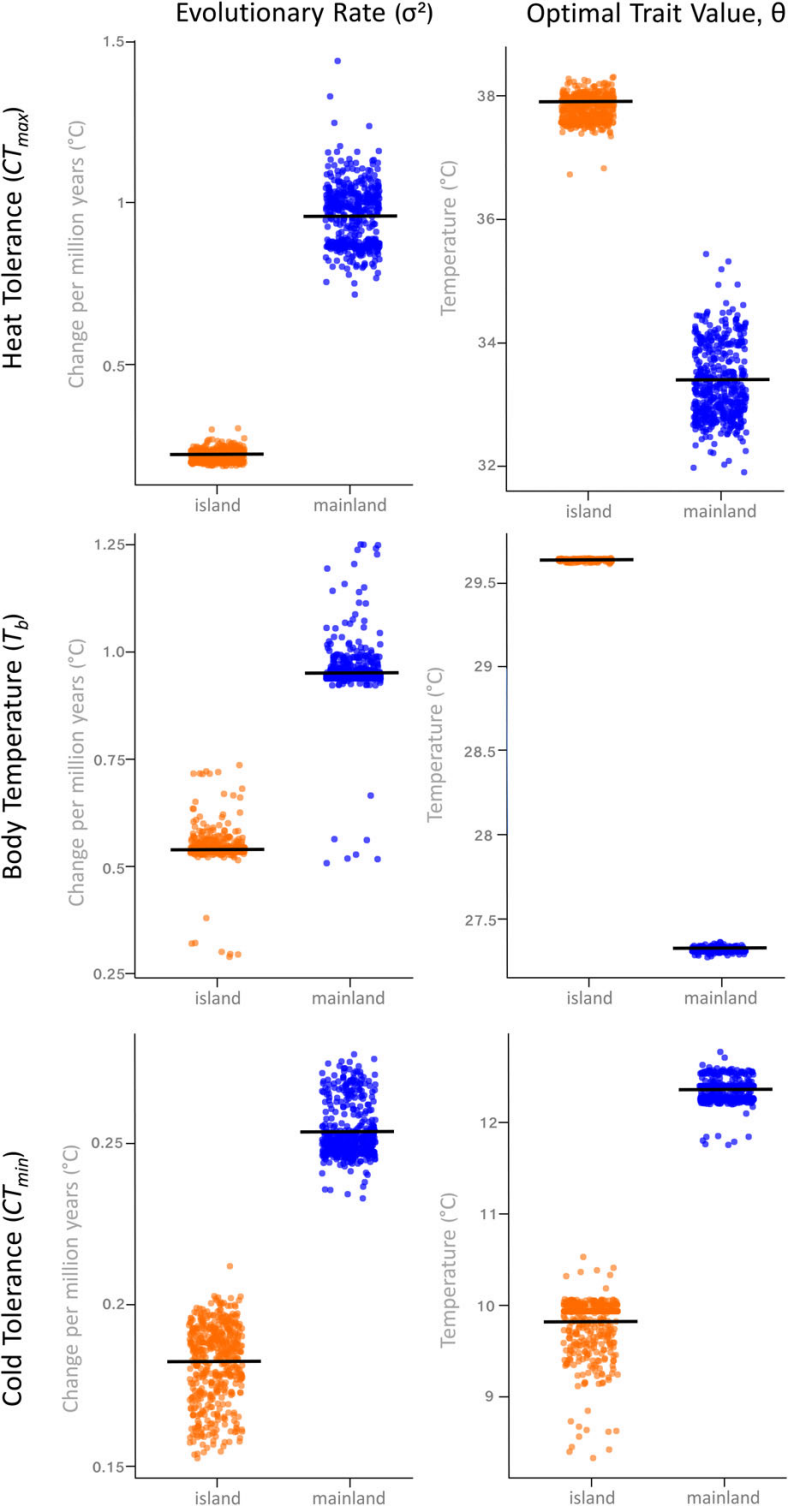
Plots summarizing the estimated rates of evolutionary change (σ^2^), and evolutionary trait optima (θ) for island (orange) and mainland (blue) lizards. Each point represents a parameter estimate from 1 of 500 stochastic character maps. Black lines indicate means.

The best‐supported phylogenetic models for heat tolerance (*CT_max_*) indicated separate evolutionary rates for mainland and island species (equal support for the BMS and OUMV models; Table 1). These results were not consistent with the island effect, however, because heat tolerance evolves approximately four times faster in mainland habitats than on islands (Fig. 2). Phylogenetic half‐life (*t*_1/2_) was 19.2% of the total tree height for island taxa, with low stationary variance (*V_y_* = 3.86) (Table 2). Together, these suggest that *CT_max_* evolves following an OU‐like process on islands, with taxa exploring a narrow range of trait space around the phenotypic optimum. In contrast, *CT_max_* evolution is similar to a BM‐like process on the mainland, as half‐life approached the total length of the tree (83.8% of tree length) and *V_y_* was substantially higher (30.26) (Table 2). The differences in evolutionary patterns between mainland and island evolution may explain why multirate OU and BM models received equivalent support in our OUwie analyses of heat tolerance evolution.

**Table 1:** Summary of the model fits for the different evolutionary models tested in this study for each physiological trait (*CT_max_*, *T_b_*, and *CT_min_*) and climatic variable (PC 1, PC 2, and PC 3)

	BM		BMS		OU1		OUM		OUMV	
Trait	∆AIC_C_	Weight	∆AIC_C_	Weight	∆AIC_C_	Weight	∆AIC_C_	Weight	∆AIC_C_	Weight
*CT_max_*	10.1	0	**0**	**0.61**	12.4	0	11.0	0	**0.6**	**0.38**
*T_b_*	39.7	0	39.1	0	17.2	0.01	**1.57**	**0.31**	**0**	**0.69**
*CT_min_*	**0**	**0.30**	**3.7**	**0.05**	**0.4**	**0.35**	**0.3**	**0.26**	**1.6**	**0.14**
PC 1	6.3	0.03	6.8	0.02	**0**	**0.61**	**2.2**	**0.21**	**3.0**	**0.14**
PC 2	7.5	0.01	7.2	0.01	**2.1**	**0.17**	**0**	**0.48**	**0.8**	**0.33**
PC 3	12.7	0.00	9.7	0.00	**0**	**0.64**	**2.2**	**0.21**	**3.0**	**0.14**

The ∆AIC_C_ score refers to the difference between model AIC_C_ and the model with the lowest score. AIC_C_ weight refers to the relative likelihood of the model. BM is a single‐peak, single‐rate Brownian motion (BM) model. BMS is a single‐peak, two‐rate BM model. OU1 is a single‐peak, single‐rate Ornstein‐Uhlenbeck (OU) model. OUM is a two‐peak, single‐rate OU model. OUMV is a two‐peak, two‐rate OU model. Models with equivalent support (∆AIC_C_ ≤ = 4) are shown in bold.

**Table 2:** Values for phylogenetic half‐life (*t*_1/2_) and stationary variance (*V_y_*) for each physiological variable estimated for island and mainland lizards

Trait	Environment	*t*_1/2_	*V_y_*
Heat tolerance (*CT_max_*)	Island	9.93	3.86
	Mainland	43.15	30.26
Body temperature (*T_b_*)	Island	16.47	5.97
	Mainland	24.48	10.51
Cold tolerance (*CT_min_*)	Island	7.18	4.05
	Mainland	8.46	7.83

The evolution of body temperature (*T_b_*) was best represented as a two‐peak OU model in which mainland and island species evolve toward distinct optima (27.2°C for mainland species and 29.5°C for island species; Table 1, Fig. 2). *t*_1/2_ and *V_y_* were both higher in mainland taxa, indicating that the rate of adaptation to the estimated optimal *T_b_* value in mainland species is weaker, perhaps reflecting additional biological factors beyond the predictors used here that drive trait evolution (Table 2). As with *CT_max_*, our results for *T_b_* indicated that mainland species explore a greater range of trait space, but do so about a lower phenotypic optimum. We found that using the reduced *T_b_* dataset (containing species with ≥10 observations) did not change the results; OUM and OUMV remained the two most strongly‐supported models for body temperature evolution (Table S4).

Support was roughly equivalent among the five models for the evolution of cold tolerance (*CT_min_*), indicating no strong support for any model more complex than a single‐peak BM (Table 1, Fig. 2). *t*_1/2_ was almost identical among island and mainland lizards, although *V_y_* was slightly in mainland species (Table 2). Together, these results suggest that the evolutionary dynamics of cold tolerance are not influenced by landmass. However, sample size for cold tolerance from island taxa was relatively low (*n* = 10), which may limit our ability to accurately detect differences between mainland and island anoles.

We did not find any evidence that mean thermal niches differ between island and mainland environments (Table S5). We also did not find that climatic niches evolve at different rates or to different optimal values between mainland and island lizards (Table 1). For PC1, PC 2, and PC3, a single‐peak OU fit the data best (or as well) as more complex OU models. To translate θ into more interpretable units, we reran the OUwie analysis using the OU1 model for MAT. When we did so, we found that optimal MAT for mainland and island habitats under a single‐peak OU was 23.6°C.

### TESTING FOR BODY TEMPERATURE‐ENVIRONMENT CORRELATIONS BETWEEN LANDMASSES

**Figure 3:**
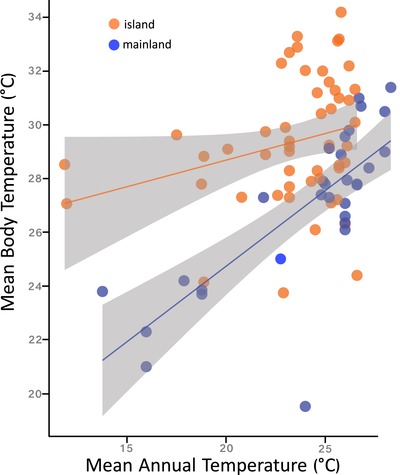
Relationship between species body temperature and mean annual temperature for mainland (blue) and island (orange lizards), showing 95% confidence bands. Each point represents a different species of *Anolis* lizard.

The best‐fitting model included PC 1 (partial correlation coefficient ± 1 SE = 0.06 ± 0.02, *P* = 0.015) and landmass (partial correlation coefficient ± 1 SE = −3.50 ± 1.20, *P* = 0.005), indicating that the relationship between body temperature and climatic environment differed between mainland and island lizards (Fig. 3). Neither PC 2 nor PC 3 were significant predictor variables of body temperature. Because MAT strongly loaded with PC 1, we reran the PGLS using MAT and landmass as predictor variables to put the relationship between local environment and body temperature into more interpretable units (data provided in Table S6). That model (*T_b_* ∼ MAT + landmass) predicts that a 1°C increase in MAT results in a 0.31°C increase in body temperature, and a transition from island to mainland results in a decrease of 2.6°C. Correspondingly, the correlation between body temperature and PC 1 was much higher in mainland lizards (*r* = 0.731, *P* = 9.8 × 10^−6^) than in island lizards (*r* = 0.124, *P* = 0.449).

## Discussion

### IS THERE AN “ISLAND EFFECT” IN THE PHYSIOLOGICAL EVOLUTION OF ANOLES?

Far from there being an island effect, we discovered that heat tolerance evolution is substantially faster on the mainland than on islands (Fig. 2). Faster rates of evolution appear to relate, at least in part, to greater stationary variance in mainland lineages, suggesting a weaker pull (if any) to a central optimum (Table 2). As a consequence, mainland anoles appear to “explore” evolutionary trait space to a greater extent than island taxa, particularly with regard to heat tolerance and body temperature. Rates of evolution were similar among landmasses for both body temperature and cold tolerance. Together, our results illustrate that physiological evolution is just as fast or even faster on mainland habitats, whereas morphological characters appear to evolve more rapidly on islands (Caetano and Harmon 2018, but see Pinto et al. 2008). A hallmark of the adaptive radiation of Caribbean anoles is rapid phenotypic divergence (Losos et al. 1998; Losos 2009). But, as physiological and morphological evidence are stitched together, it is clear that mainland habitats provide as much, if not more, opportunity for phenotypic diversification as islands, and that the adaptive radiation of anoles is not restricted to insular habitats.

Why might physiological evolution be faster in mainland anoles? One possible explanation is that climatic niches evolve faster in mainland habitats than on islands (i.e., greater climatic niche turnover; Velasco et al. 2018), and that patterns of physiological evolution are correlated with climatic niche shifts (e.g., Kozak and Wiens 2010; Castro‐Insua et al. 2018). However, we found that rates of thermal niche evolution were similar between island and mainland lineages, suggesting that this is not the case (Table 1).

Behavioral thermoregulation noticeably differs between landmasses, with both slope and intercept of *T_b_‐*environment relationships varying between mainland and island habitats (Table 2, Fig. 3). Specifically, body temperature was positively correlated with thermal environment in mainland anoles, such that montane species had core temperatures more than 10˚C cooler than their lowland counterparts (Fig. 3). In contrast, body temperature in island lizards remained substantially more stable, even at higher elevations where environments were substantially cooler. These behavioral differences between island and mainland anoles may, in turn, impart distinct footprints on physiological evolution. The Bogert effect, or “behavioral inertia,” occurs when organisms are shielded from selection through behavioral buffering, thus forestalling physiological adaptation to local conditions (Huey et al. 2003). More stable body temperatures in island species are consistent with the slower rates of heat tolerance evolution and higher optimal body temperatures that we observed (Fig. 2). As such, the macroevolutionary signature of the Bogert effect appears to be stronger on islands than on the mainland.

Given that thermal heterogeneity (i.e., temperature variation in a habitat) is often quite high during the day, thermoregulation and the Bogert effect can be particularly effective on upper physiological limits (Sunday et al. 2011; Leal and Gunderson 2012; Muñoz et al. 2014, 2016; Buckley et al. 2015). The ability for organisms to thermoregulate during the night, however, is much more limited because temperatures become much more stable and progressively cooler with elevation (Sarmiento 1986; Ghalambor et al. 2006; Muñoz and Bodensteiner 2019). In contrast to heat tolerance and body temperature, cold tolerance evolution showed no biogeographic pattern, as both rates and optimal trait values were similar between island and mainland lizards. Without behavioral refuges from the cold, montane lizards on both mainland and island habitats may have no option but to adjust their physiology (Muñoz et al. 2014; Muñoz and Bodensteiner 2019).

### ECOLOGICAL OPPORTUNITY AND PATTERNS OF PHYSIOLOGICAL EVOLUTION

Although the Bogert effect can help explain why patterns of physiological evolution differ, it cannot explain why body temperature patterns should vary between mainland and island habitats. One possibility is that the distribution of thermal patches within a given habitat may be more conducive to thermoregulation on islands than on the mainland. Thermoregulatory ability is contingent on how the thermal landscape is structured (Sears and Angilletta 2015; Sears et al. 2016). Two habitats with the same macroclimatic conditions (such as MAT) may be structured in different ways at a finer scale (e.g., Hertz 1992). For example, the distribution of thermal patches may be more “coarse‐grained” in a dense forest where closed canopy creates large swaths of thermally homogenous habitat, or be more “fine‐grained” along forest edges where abundant shifts in shade/sun structure create more thermally heterogeneous habitat. Behaviorally maintaining a relatively high core temperature can be beneficial because maximal performance (e.g., sprint speed) is often positively correlated with temperature (Angilletta et al. 2002, 2009). However, shuttling between preferred thermal microclimates imposes costs, and when transit distances are high those costs magnify and potentially outweigh the benefits (Huey 1974; Huey and Slatkin 1976; Vickers et al. 2011; Sears and Angilletta 2015). Addressing how thermal structure impacts behavior is feasible, but it would require fine‐scale sampling of thermal habitats using an appropriate null model approach (e.g., Hertz et al. 1993), and a deeper understanding of the vegetation structure that anoles use on islands and the mainland.

Mainland and island anoles may interact differently with their thermal environments, reflecting the distinct selective pressures these lizards experience. Mainland predators are more diverse than island predators (Greene 1988; Henderson and Crother 1989) and anole mortality rates are higher on the mainland (Andrews 1979; McLaughlin and Roughgarden 1989). Correspondingly, mainland anoles spend considerably less time moving around their habitats than island species, and are generally more cryptic in their behavior (Perry 1999; Irschick et al. 2000; Cooper 2005; Johnson et al. 2008; Losos 2009). In fact, mainland anoles can spend most of their time hiding in refuges rather than actively moving around their habitats (Lister and Garcia Aguayo 1992). Movement between patches is a key factor in spatially explicit models of thermoregulation (Sears and Angilletta 2015). In addition to stronger predation, mainland anoles also experience stronger competition, due both to a greater number of anole species and other lizards (discussed in Losos 2009). As with predation, stronger competition may also constrain movement rates (e.g., Kamath and Stuart 2015). Even if the thermal landscapes were equivalent (or nearly so) among landmasses, stronger predation pressure should increase the fitness costs of thermoregulation (Huey 1974; Huey and Slatkin 1976). Increased predation and competition may result in mainland lizards being more behaviorally passive with regard to thermal environment, and help explain the strong positive relationship we observed between *T_b_* and thermal environment (Fig. 3).

Release from predation and competition is one of the key defining features of ecological opportunity, which has been often invoked to explain the extraordinary morphological diversity of Caribbean anoles (Losos 2010; Mahler et al. 2010; Stroud and Losos 2016) and other adaptive radiations (e.g., Schluter 2000). We suggest that ecological opportunity may not always facilitate evolution. In the case of island anoles, ecological opportunity may, in fact, indirectly result in slower physiological evolution. Specifically, release from predators and competitors may allow island species to exploit their thermal habitats more freely than mainland species, due to the lower extrinsic costs of thermoregulation. As a result, island species may capitalize on the fitness benefits of a higher core temperature such as a higher maximum sprint speed and faster digestion rate (Huey and Kingsolver 1989; Angilletta et al. 2009), resulting in a slower physiological evolution on islands. As such, the Bogert effect may be an important, but less appreciated aspect of island diversification (Muñoz and Losos 2018). As sampling of anole behavior, physiology, and thermal habitats continue to increase, this idea can be more rigorously tested. We note that the generalities stated here might not apply to all cases. For example, some island species are known to be behaviorally passive, allowing their body temperature to fluctuate with local conditions (e.g., Huey and Webster 1975), and thermoregulatory patterns can vary spatially across a species’ range (Huey 1983). Thus, patterns of physiological evolution and thermal behavior on islands may be more nuanced than we are able to currently assess. We further note that our understanding of evolutionary trait dynamics will continue to improve as more physiological data become available. For example, low species numbers for cold tolerance (particularly from islands) may have limited our ability to contrast evolutionary patterns.

### CONCLUDING REMARKS AND IMPLICATIONS FOR ADAPTIVE RADIATION

Faster physiological evolution in mainland anoles underscores that the adaptive radiation of anoles encompasses both mainland and island habitats. In a similar vein, other researchers have found that mainland radiations also have repeatable “ecomorph” communities among habitats (Moreno‐Arias and Calderón‐Espinosa 2016). Because lower predation rates should lower the costs for thermoregulation, then release from selection on islands might indirectly dampen physiological evolution. As such, ecological opportunity may indirectly stymie physiological evolution through the Bogert effect. Given that ecological opportunity is such an important factor underlying adaptive radiation (Schluter 2000; Losos 2009; Mahler et al. 2010), its indirect evolutionary consequences on physiology may potentially be widespread, although generally underexplored.

At a broader level, our results highlight how physiological and morphological traits can exhibit distinct patterns of diversification during adaptive radiation. This finding is broadly relevant beyond *Anolis* lizards. For example, adaptive radiation along depth gradients in aquatic habitats should impact numerous variables such as light environment, prey communities, available oxygen, and temperature. In other words, diversification along any resource axis should simultaneously impinge on numerous distinct and potentially contrasting selection pressures (Lewontin 1983; Levins and Lewontin 1985; Huey et al. 2003). To the extent that selection pressures covary along phenotypic axes, we might predict similar patterns of trait evolution. In contrast, if the effects of behavior on selection differ among traits (e.g., morphology vs. physiology) or among habitats (e.g., mainland vs. islands), then patterns of phenotypic evolution may be quite different. Detailed studies linking behavior, physiology, and morphology in a broader biogeographic framework will more deeply reveal the factors and interactions that mold exceptional patterns of phenotypic diversity in nature.

## CONFLICT OF INTEREST

The authors declare that they have no conflict of interest.

Associate Editor: A. Crawford

Handling Editor: P. Tiffin

## Supporting information


**Table S1**. Summary of the thermal data we used in the analyses. Mean species’ values for *CT_min_*, *T_b_*, and *CT_max_* are given in °C. Numbers in parentheses refer to the sample size (*N*) for each trait.
**Table S2**. Description of thermal bioclim variables used in this study.
**Table S3**. Loadings and eigenvalues from a phylogenetic PC analysis on the thermal climate variables from the WorldClim database. Strong loadings shown in bold. Definition of each variable given in Table S2.
**Table S4**. Summary of the model fits for the different evolutionary models tested in this study for each body temperature (*T_b_*) in the dataset limited to species with ≥ 10 individuals.
**Table S5**. Results of phylogenetic ANOVAs comparing climatic PC variables and several bioclim variables between mainland and island habitats.
**Table S6**. Summary of the body temperature and environmental (mean annual temperature) data we used in the thermoregulatory analyses.

## Data Availability

All data used for analyses in this manuscript are included in the Supplementary Materials.
