# Supplementary material for: Physiological evolution during adaptive radiation: A test of the island effect in Anolis lizards
Source: Evolution. 2019 Apr 23;73(6):1241–52. doi: 10.1111/evo.13741 (PMC6593988; doi:10.1111/evo.13741)
Supplement: Supplementary file 1 — Table S1. Summary of the thermal data we used in the analyses. Mean species’ values for CTmin, Tb, and CTmax are given in °C. Numbers in parentheses refer to the sample size (N) for each trait. Table S2. Description of thermal bioclim variables used in this study. Table S3. Loadings and eigenvalues from a phylogenetic PC analysis on the thermal climate variables from the WorldClim database. Strong loadings shown in bold. Definition of each variable given in Table S2. Table S4. Summary of the model fits for the different evolutionary models tested in this study for each body temperature (Tb) in the dataset limited to species with ≥ 10 individuals. Table S5. Results of phylogenetic ANOVAs comparing climatic PC variables and several bioclim variables between mainland and island habitats. Table S6. Summary of the body temperature and environmental (mean annual temperature) data we used in the thermoregulatory analyses. [file EVO-73-1241-s001.docx]

**Table S1.** Summary of the thermal data we used in the analyses. Mean species’ values for *CT_min_*, *T_b_*, and *CT_max_* are given in °C. Numbers in parentheses refer to the sample size (*N*) for each trait.

| *Anolis* species | Origin | *CT_min_* (*N*) | *T_b_* (*N*) | *CT_max_* (*N*) | Reference |
| --- | --- | --- | --- | --- | --- |
| *acutus* | Island | - | 30.92 (131) | 39.5 (7) | McManus and Nellis 1973; Ruibal and Philibosian 1974; Gunderson et al 2018 |
| *aeneus* | Island | - | 31.00 (91) | - | Schoener and Gorman 1968; Roughgarden et al. 1981 |
| *aequatorialis* | Mainland | - | 22.30 (2) | - | Fitch et al. 1976 |
| *agassizi* | Island | - | 30.60 (40) | - | Rand et al. 1975 |
| *aliniger* | Island | - | 28.83 (3) | - | B. Bodensteiner et al., unpubl. data |
| *allisoni* | Island | - | 32.61 (240) | - | Ruibal 1961; Logan et al. 2013; Medina et al. 2016 |
| *allogus* | Island | - | 29.2 (148) | - | Ruibal 1961 |
| *alutaceus* | Island | - | 28.90 (7) | - | Rodríguez-Schettino et al. 2010 |
| *antonii* | Mainland | 13.11 (45) | - | 28.12 (49) | J.C. Salazar et al., unpubl. Data |
| *angusticeps* | Island |  | 29.2 (3) |  | Rodríguez-Schettino et al. 2010 |
| *apletophallus* | Mainland | - | 27.80 (30) | - | Ballinger et al. 1970; Stapley et al. 2015 |
| *armouri* | Island | 7.63 (21) | 28.19 (111) | 39.30 (9) | Muñoz et al. 2014; Conover et al. 2015; Muñoz and Losos 2018 |
| *auratus* | Mainland | - | - | 40.00 (17) | Ballinger et al. 1970 |
| *bahorucoensis* | Island | - | 24.76 (20) | - | Cast et al. 2000 |
| *baleatus* | Island | - | 28.50 (2) | - | B. Bodensteiner et al., unpubl. data |
| *barbatus* | Island | - | 27.3 (3) | - | Rodríguez-Schettino et al. 2010 |
| *barbouri* | Island | - | 27.31 (8) | - | B. Bodensteiner et al., unpubl. data |
| *barkeri* | Mainland | - | 24.02 (72) | - | Birt et al. 2001 |
| *bartschi* | Island | - | 26.10 (115) | - | Estrada and Novo 1987 |
| *bicaorum* | Island | - | 29.9 (142) | - | Logan et al. 2013 |
| *bimaculatus* | Island | - | 32.90 (30) | - | Roughgarden et al. 1981 |
| *biscutiger^[[1]](#footnote-1)^* | Mainland | - | 31.00 (36) | - | Fitch et al. 1976 |
| *bonariensis* | Island | - | 33.40 (31) | - | Bennett and Gorman 1979 |
| *brasiliensis* | Mainland | - | 31.62 (102) | - | Mesquita et al. 2015 |
| *brevirostris* | Island | - | 30.60 (54) | - | Moster et al. 1992 |
| *capito* | Mainland | - | 28.90 (16) | - | Vitt and Zani 2005 |
| *carolinensis* | Mainland | 10.15 (15) | 23.70 (448) | 43.08 (130) | Lister 1976; Wilson and Echternacht 1987; Wilson and Echternacht 1990; Jenssen et al. 1996; Kolbe et al. 2012; Rubio 2012 |
| *carpenteri* | Mainland | - | 27.30 (1) | - | Fitch et al. 1976 |
| *chloris* | Mainland | 12.57 (12) | - | 32.16 (7) | J.C. Salazar et al., unpubl. data |
| *chlorocyanus* | Island | - | 30.41 (11) | - | B. Bodensteiner et al., unpubl. data |
| *christophei* | Island | - | 24.15 (8) | - | B. Bodensteiner et al., unpubl. data |
| *chrysolepis* | Mainland | 9.40 (19) | - | 39.7 (19) | Diele-Viegas et al. 2018 |
| *coelestinus* | Island | - | 28.70 (28) | - | Sifers et al. 2001; B. Bodensteiner et al., unpubl. data |
| *cooki* | Island | 9.30^1^ | 32.03 (516) | 38.80^1^ | Lister 1976; Hertz 1992; Huey unpub. Data; Huey et al. (2009) |
| *cristatellus* | Island | 9.72 (46) | 27.38 (657) | 39.00 (10) | Lister 1976; Hertz 1992; Hertz 1992; Kolbe et al. 2012; Leal and Gunderson 2012 |
| *cupreus* | Mainland | 11.81 (19) | 29.4 (57) | 38.40 (19) | van Berkum 1986; van Berkum 1988 |
| *cuprinus* | Mainland | - | 30.70 (24) | - | Fitch et al. 1976 |
| *cuvieri* | Island | - | - | 35.0 (3) | Gunderson et al. 2018 |
| *cybotes* | Island | 10.66 (85) | 29.66 (602) | 39.41 (89) | Hertz and Huey 1981; Cast et al. 2000; Muñoz et al. 2014; Conover et al. 2015 |
| *distichus* | Island | - | 29.26 (87) | - | Lister 1976; Cast et al. 2000 |
| *dollfusianus* | Mainland | - | 28.40 (31) | - | Fitch et al. 1976 |
| *eulaemus* | Mainland | 11.50 (1) | - | 23.60 (1) | J.C. Salazar et al., unpubl. data |
| *evermanni* | Island | - | 25.90 (48) | 36.9 (10) | Rand 1964; Hertz 1977; Gunderson et al. 2018 |
| *frenatus* | Mainland | - | - | 35.70 (8) | Ballinger et al. 1970 |
| *fuscoauratus* | Mainland | 8.40 (48) | 28.60 (86) | 39.80 (46) | Diele-Vargas et al. (2018) |
| *gadovii* | Mainland | - | 30.50 (1) | - | Fitch et al. 1976 |
| *garmani* | Island | - | - | 38.8 (10) | Gunderson et al. 2018 |
| *gemmosus* | Mainland | - | 21.00 (34) | - | Fitch et al. 1976 |
| *gingivinus* | Island | - | 30.10 (315) | - | Eaton et al. 2002 |
| *grahami* | Island | - | 31.20 (85) | 39.3 (10) | Rand 1967; Lister 1976; Gunderson et al. 2018 |
| *granuliceps* | Mainland | 15.50 (2) | - | 32.69 (1) | J.C. Salazar et al., unpubl. data |
| *gundlachi* | Island | 6.30^[[2]](#footnote-2)^ | 23.75 (1415) | 35.40^1^ | Hertz 1981; Hertz 1992; Huey and Webster 1976; Huey unpub. Data; Huey et al. (2009) |
| *heterodermus* | Mainland | 6.73 (4) | 23.8 (98) | 31.51 (5) | Méndez-Galeano and Calderón-Espinosa 2017; J.C. Salazar et al., unpubl. Data |
| *homolechis* | Island | - | 31.8 (104) | - | Ruibal 1961 |
| *humilis* | Mainland | 12.40 (22) | 26.2 (110) | 35.62 (22) | vanBerkum 1986; vanBerkum 1988 |
| *jubar* | Island | - | 32.40^[[3]](#footnote-3)^ | - | Hertz et al 2013; J.B. Losos et al., unpubl. data |
| *krugi* | Island | - | 28.6 (39) | 37.9 (10) | Rand 1964; Gunderson et al. 2018 |
| *latifrons* | Mainland | 12.50 (2) | - | 36.12 (2) | J.C. Salazar et al., unpubl. Data |
| *laeviventris^[[4]](#footnote-4)^* | Mainland | 11.06 (8) | 25.96 (191) | 38.44 (8) | Fitch 1972; vanBerkum 1986; vanBerkum 1988 |
| *lemurinus* | Mainland | 12.90 (9) | 28.8 (202) | 37.30 (9) | van Berkum 1986; van Berkum 1988; Logan et al. 2013 |
| *limifrons* | Mainland | 12.32 (14) | 26.55 (343) | 34.33 (51) | Ballinger et al. 1970; Fitch 1973; vanBerkum 1986; vanBerkum 1988 |
| *lineatopus* | Island | - | 28.30 (101) | 35.8 (10) | Lister 1976; Gunderson et al. 2018 |
| *lionotus* | Mainland | 14.72 (9) | 26.40^[[5]](#footnote-5)^ | 36.73 (9) | Campbell 1971; vanBerkum 1986; vanBerkum 1988 |
| *longiceps* | Island | - | 32.20 (18) | - | Powell 1999 |
| *longitibialis* | Island | 12.80 (18) | 29.21 (105) | 38.50 (18) | Hertz and Huey 1981; Muñoz et al. 2014 |
| *loysianus* | Island |  | 29.02 (5) |  | Rodríguez-Schettino et al. 2010 |
| *lucius* | Island | - | 29.3 (86) | - | Ruibal 1961 |
| *luteogularis* | Island |  | 29.4 (8) |  | Rodríguez-Schettino et al. 2010 |
| *lyra* | Mainland | 17.86 (1) | - | 36.50 (1) | J.C. Salazar et al., unpubl. data |
| *maculigula* | Mainland | 13.10 (12) | - | 29.58 (8) | J.C. Salazar et al., unpubl. data |
| *maculiventris* | Mainland | 18.17 (9) | - | 31.86 (8) | J.C. Salazar et al., unpubl. data |
| *marcanoi* | Island | 11.77 (25) | 29.10 (48) | 38.52 (25) | Muñoz et al. 2014 |
| *marmoratus* | Island | - | 29.75 (638) | - | Huey and Webster 1975 |
| *mestrei* | Island | - | 27.70^[[6]](#footnote-6)^ | - | P.E. Hertz, unpubl. Data; Hertz et al 2013 |
| *monensis* | Island | - | 31.29 (245) | - | Lister 1976 |
| *nebulosus* | Mainland | - | 30.01 (1697) | - | Ramírez-Bautista and Benabib 2001 |
| *notopholis* | Mainland | 14.43 (2) | - | 32.41 (2) | J.C. Salazar et al., unpubl. data |
| *oculatus* | Island | - | 28.01 (257) | 34.50 (9) | Brooks 1968; Malhotra and Thorpe 1993; *Hertz 1979* |
| *olssoni* | Island | - | 32.55 (76) | - | Smith et al. 1994; Hertz 1979a |
| *opalinus* | Island | - | 28.10 (11) | 37.00 (10) | Rand 1967; Gunderson et al. 2018 |
| *cf. orcesi* | Mainland | 13.48 (3) | - | 28.23 (2) | J.C. Salazar et al., unpubl. data |
| *ortonii* | Mainland | 9.70 (4) | 30.30 (7) | 42.30 (3) | Diele-Viegas et al. 2018 |
| *oxylophus* | Mainland | - | 27.77 (54) | - | Vitt et al. 1995 |
| *planiceps* | Mainland | 9.60 (11) | 28.30 (19) | 40.30 (11) | Diele-Viegas et al. 2018 |
| *poecilopus* | Mainland | - | 26.50^4^ | - | Campbell 1971 |
| *polylepis* | Mainland | - | 27.51 (42) | - | Hertz 1974 |
| *poncensis* | Island | - | 32.60 (26) | 41.10 (11) | Rand 1964; Gunderson et al. 2018 |
| *porcatus* | Island | - | 32.7 (3) | - | Rodríguez-Schettino et al. 2010 |
| *princeps* | Mainland | - | 27.40 (1) | - | Fitch et al. 1976 |
| *proboscis* | Mainland | - | 23.85 (2) | - | Losos et al. 2012 |
| *pulchellus* | Island | - | 32.60 (44) | 35.69 (17) | Rand 1964; Hertz 1979b |
| *punctatus* | Mainland | 8.00 (2) | 29.2 (32) | 39.6 (2) | Vitt et al. 2003b; Diele-Viegas et al. 2018 |
| *purpurescens^[[7]](#footnote-7)^* | Mainland | 18.97 (2) | - | 31.97 (2) | J.C. Salazar et al., unpubl. data |
| *quercorum* | Mainland | - | 24.40^4^ | - | Fitch 1978 |
| *richardi* | Island | - | 28.40 (39) | - | Roughgarden et al. 1981 |
| *roquet* | Island | - | 27.22 (508) | 35.66 (42) | Hertz 1981 |
| *sagrei* | Island | - | 32.01 (1013) | 41.9 (10) | Ruibal 1961; Lister 1976; Lee 1980; Kolbe et al. 2012; Rubio 2012; Gunderson et al. 2018 |
| *scypheus* | Mainland | - | 27.30 (36) | - | Avila-Pires 1995; Diele-Viegas et al. 2018 |
| *semilineatus* | Island | - | 31.85 (32) | 38.34 (29) | Hertz 1979a; Hertz 1979b |
| *sericeus* | Mainland | - | 32.80 (15) | - | Fitch 1973 |
| *shrevei* | Island | 7.73 (20) | 29.05 (130) | 39.73 (20) | Hertz and Huey 1981; Muñoz et al. 2014; Muñoz and Losos 2018 |
| *singularis* | Island | - | 29.63 (3) | - | B. Bodensteiner et al., unpubl. data |
| *smaragdinus* | Island | - | 34.20 (71) | - | Lister 1976 |
| *strahmi* | Island | 11.30 (6) | 28.04 (10) | 39.20 (6) | Muñoz et al. 2014; B. Bodensteiner et al., unpubl. data |
| *stratulus* | Island | - | 28.40 (63) | 39.20 (9) | Rand 1964; Gunderson et al. 2018 |
| *subocularis* | Mainland | - | 31.40 (13) | - | Fitch et al. 1976 |
| *tandai* | Mainland | - | 27.70 (33) | - | Vitt et al. 2001 |
| *taylori* | Mainland | - | 29.10 (28) | - | Fitch et al. 1976 |
| *townsendi* | Island | - | 30.90 (24) | - | Carpenter 1965 |
| *trachyderma* | Mainland | - | 27.80 (1) | - | Vitt et al. 2003a; Diele-Vargas et al. 2018 |
| *transversalis* | Mainland | - | 29.00 (12) | - | Diele-Vargas et al. 2018 |
| *tropidogaster* | Mainland | - | - | 33.40 (4) | Ballinger et al. 1970 |
| *tropidolepis* | Mainland | 9.49 (11) | 19.52 (320) | 33.06 (11) | VanBerkum 1986; vanBerkum 1988 |
| *uniformis* | Mainland | - | 27.90 (19) | - | Birt et al. 2001 |
| *valencienni* | Island | - | - | 40.5 (6) | Gunderson et al. 2018 |
| *ventrimaculatus* | Mainland | 12.52 (35) | - | 27.02 (54) | J.C. Salazar et al., unpub. data |
| *vermiculatus* | Island | - | 29.71 (16) | - | Rodríguez-Schettino et al. 2010 |
| *wattsi* | Island | - | 33.30 (17) | - | Roughgarden et al. 1981 |
| *whitemani* | Island | 12.20 (15) | 27.90 (17) | 38.80 (15) | Muñoz et al. 2014 |
| *woodi^[[8]](#footnote-8)^* | Mainland | - | 21.50 (1) | - | Fitch et al. 1976 |

**Work Cited**

Avila-Pires, T. C. S. 1995. Lizards of Brazilian Amazonia (Reptilia: Squamata). Zoologische erhandelingen. 299(1):706.

Ballinger, R. E., K. R. Marion and O. J. Sexton. 1970. Thermal ecology of the lizard, *Anolis limifrons* with comparative notes on three additional Panamanian anoles. Ecology. 51(2): 246-254.

Bennett, A. F. and G. C. Gorman. 1979. Population density and energetics of lizards on a tropical island. Oecologia. 42:339-358.

Birt, R. A., R. Powell and B. D. Greene. 2001. Natural history of *Anolis barkeri*: A semiaquatic lizard from Southern México. Journal of Herpetology. 35(1):161-166.

Brooks, G. R. 1968. Body temperatures of three lizards from Dominica, West Indies. Herpetologica 24:209–214.

Campbell, H. 1971. Observations on the thermal activity of some tropical lizards of the genus *Anolis* (Iguanidae). Carib. J. Sci. 11:17–20.

Carpenter, C. C. 1965. The display of the Cocos Island anole. Herpetologica. 21:256–260.

Cast, E. E., M. E. Gifford, K. R. Schneider, A. J. Hardwick, J. S. Parmerlee, Jr., and R. Powell. 2000. Natural history of an anoline lizard community in the Sierra de Baoruco, Dominican Republic. Caribbean Journal of Science. 36:258–266.

Conover, A. E., G. Ellee. K. E. Cook, K. E. Boronow and M. M. Muñoz. 2015. Effects of ectoparasitism ob behavioral thermoregulation in the tropical lizards *Anolis cybotes* (Squamata: Dactyloidae) and *Anolis armouri* (Squamata: Dactyloidae). Breviora. 545(1)1-13.

Diele-Viegas, L. M., L. J. Vitt, B. Sinervo, G. R. Colli, F. P. Werneck, D. B. Miles, W. E. Magnusson, J. C. Santos, C. M. Sette, G. H. O. Caetano, E. Pontes, T. C. S. Ávila-Pires. 2018. Thermal physiology of Amazonian lizards (Reptilia: Squamata). PLoS ONE 13(3): e0192834.

Eaton, J. M., S. C. Larimer, K. G. Howard, R. Powell, and J. S. Parmerlee, Jr. 2002. Population densities and ecological release of a solitary species: *Anolis gingivinus* on Anguilla, West Indies. Caribbean Journal of Science. 38:27–36.

Estrada, A. R., and J. Novo. 1987. Subnicho climático de *Anolis bartschi* (Sauria: Iguanidae). Poeyana. 341:1–19.

Fitch, H. S. 1972. Ecology of *Anolis tropidolepis* in Costa Rican cloud forest. Herpetologica. 28:10-21.

Fitch, H. S. 1973. A field study of Costa Rican lizards. University of Kansas Science Bulletin. 50:39–126.

Fitch, H. S. 1978. Two new anoles (Reptilia: Iguanidae) from Oaxaca with comments on other Mexican species. Contributions in biology and geology, Milwaukee Public Museum No. 20:1–15.

Fitch, H. S., A. F. Echelle, and A. A. Echelle. 1976. Field observations on rare or little known mainland anoles. University of Kansas Science Bulletin. Bull 51:91–128.

González Bermúdez, F., and L. Rodríguez Schettino. 1982. Datos etoecológicos sobre *Anolis vermiculatus.* Poeyana. 245:1–18.

Gunderson, A. E., L. Mahler and M. Leal. 2018. Thermal niche evolution across replicated *Anolis* lizard adaptive radiations. Proceedings of the Royal Society, B. 285: 20172241.

Hertz, P. E. 1974. Thermal passivity of a tropical forest lizard, *Anolis polylepis*. Journal of Herpetology. 8:323–327.

Hertz, P. E. 1977. Altitudinal variation in thermoregulatory strategies, physiological ecology, and morphology of some West Indian anoles. Doctoral dissertation, Harvard University, Cambridge, MA.

Hertz, P. E. 1979a. Comparative thermal biology of sympatric grass anoles (*Anolis semilineatus* and *A. olssoni*) in lowland Hispaniola (Reptilia, Lacertilia, Iguanidae). Journal of Herpetology. 13:329–333.

Hertz 1979b. Sensitivity to high temperature in the West Indian Grass anoles (Sauria, Iguanidae), with a review of heat sensitivity in the genus *Anolis*. Comp. Biochem. Physiol. 63A:217-222.

Hertz, P. E. 1981. Adaptation to altitude in two west Indian anoles (Reptilia: Iguanidae): field thermal biology and physiological ecology. Journal of Zoology of London. 195:25–37.

Hertz, P. E. 1992. Evaluating thermal resource partitioning by sympatric lizards *Anolis cooki* and *A. cristatellus*: a field test using null hypotheses. Oecologia. 90:127–136.

Hertz, P. E., Y. Arima. A. Harrison, R. B. Huey. J. B. Losos and R. E. Glor. 2013. Asynchronous evolution of physiology and morphology in *Anolis* lizards. Evolution. 67(7):2101-2113.

Hertz, P. E., and R. B. Huey. 1981. Compensation for altitudinal changes in the thermal environment by some *Anolis* lizards on Hispaniola. Ecology. 62:515–521.

Huey, R. B., and T. P. Webster. 1975. Thermal biology of a solitary lizard: *Anolis marmoratus* of Guadeloupe, Lesser Antilles. Ecology 56:445–452.

Jenssen, T. A., J. D. Congdon, R. U. Fischer, R. Estes, D. Kling, S. Edmands, and H. Berna. 1996. Behavioural, thermal, and metabolic characteristics of a wintering lizard (*Anolis carolinensis*) from South Carolina. Functional Ecology. 10:201–209.

Kolbe, J. J., P.S. VanMiddlesworth, N. Losin, N. Dappen and J. B. Losos. 2012. Climatic niche shift predicts thermal trait response in one but not both introductions of the Puerto Rican lizard *Anolis cristatellus* to Miami, Florida, USA. Ecology and Evolution. 2(7):1503–1516.

Leal, M. and A. R. Gunderson. 2012. Rapid change in the thermal tolerance of a tropical lizard. American Society of Naturalist. 180(6):815-822.

Lister, B. C. 1976. The nature of niche expansion in West Indian *Anolis* lizards I: ecological consequences of reduced competition. Evolution. 30:659–676.

Logan, M. L., Huynh, R. K., Precious, R. A., and R. G. Calsbeek. 2013. The impact of climate change measured at relevant spatial scales: new hope for tropical lizards. Global Change Biology. 19:3093-3102.

Losos, J. B., M. L. Woolley, D. L. Mahler, O. Torres.Carvajal, K. E. Crandell, E. W. Schaad, A. E. Narváez, F. Ayala-Varela and A. Herrel. 2012. Notes on the Natural History of the Little-Known Ecuadorian Horned Anole, *Anolis proboscis*. Breviora. 531:1-17.

Malhotra, A., and R. S. Thorpe. 1993. An experimental field study of a eurytopic anole, *Anolis oculatus*. Journal of Zoology of London. 299:163–170.

McManus, J. J., and D. W. Nellis. 1973. Temperature and metabolism of a tropical lizard, *Anolis acutus*. Comparative Biochemistry and Physiology. 45A:403–410.

Medina, M., J. B. Fernández, P. Charruau, F. Méndez de la Cruz and N. Ibargüengoytía. 2016. Vulnerability to climate change of *Anolis allisoni* in the mangrove habitats of Banco Chinchorro Islands, Mexico. Journal of Thermal Biology. 58:8-14.

Méndez-Galeano, M. A. and M. L. Calderón-Espinosa. 2017. Thermoregulation in the Andean lizard *Anolis heterodermus* (Squamata: Dactyloidae) at high elevation in the Eastern Cordillera of Colombia. Iheringia, Série Zoologia. 107:e2017018.

Mesquita, D. O, G. C. Costa, A. S. Figueredo, F. G.R. França, A. A. Garda, A. H. Bello Soares, L. Tavares-Bastos, M. M. Vasconcellos, G. H. C. Vieira, L. J. Vitt, F. P. Werneck, H. C. Wiederhecker, and G. R. Colli. 2015. The autecology of *Anolis brasiliensis* (Squamata, Dactyloidae) in a Neotropical Savanna. Herpetological Journal. 25:233-244.

Moster, J. A., R. Powell, J. S. Parmerlee, Jr., D. D. Smith, and A. Lathrop. 1992. Natural history notes on a small population of *Anolis brevirostris* (Sauria: Polychridae) from altered habitat in the Dominican. Bulletin of the Maryland Herpetological Society. 28:150–161.

Muñoz, M. M., and J. B. Losos. 2018. Thermoregulation simultaneously promotes and forestalls evolution in a tropical lizard. American Naturalist. 191:E15-E26.

Muñoz, M. M., Stimola, M. A., Algar, A. C., Conover, A., Rodriguez, A. J., Landestoy, M. A., Bakken, G. S. and. J. B. Losos, J.B. 2014. Evolutionary stasis and lability in thermal physiology in a group of tropical lizards. Proceedings of the Royal Society, B. 281:20132433.

Powell, R. 1999. Herpetology of Navassa Island, West Indies. Caribbean Journal of Science. 35:1–13.

Ramírez-Bautista, A., and M. Benabib. 2001. Perch height of the arboreal lizard *Anolis nebulosus* (Sauria: Polychrotidae) from a tropical dry forest of Mexico: Effect of the reproductive season. Copeia. 2001:187–193.

Rand, A. S. 1964. Ecological distribution in anoline lizards of Puerto Rico. Ecology. 45:745–752.

Rand, A. S. 1967. The ecological distribution of the anoline lizards around Kingston, Jamaica. Breviora, Museum of Comparative Zoology. 272:1–18.

Rand, A. S., G. C. Gorman, and W. M. Rand. 1975. Natural history, behavior, and ecology of *Anolis agassizi.* Smithsonian Contributions to Zoology. 76:27–38.

Rodríguez-Schettino, L., Losos, J. B., Hertz, P. E., de Queiroz, K., Chamizo, A. D., Leal, M., and V. R. González. 2010. The anoles of Soroa: aspects of their ecological relationships. Breviora 520:1-22.

Roughgarden, J., W. Porter, and D. Heckel. 1981. Resource partitioning of space and its relationship to body temperature in *Anolis* Lizard populations. Oecologia. 50:256–264.

Rubio, L. C. M. 2012. Geographic variation in the lower temperature tolerance in the invasive brown anole, *Anolis sagrei* and the native green anole, *Anolis carolinensis* (sauria: polychrotidae). Doctoral dissertation, University of Tennessee, Knoxville, TN.

Ruibal, R. 1961. Thermal relations of five species of tropical lizards. Evolution. 15:98–111.

Ruibal, R., and R. Philibosian. 1974. The population ecology of the lizard *Anolis acutus*. Ecology 55:525–537.

Salazar, J. C., Castañeda, M. R., and G. A. Londoño. 2018. Intra- and interspecific variation in the critical thermal minimum and maximum of mainland anole lizards (Reptilia: Squamata: Dactyloidae: *Anolis*). Undergraduate dissertation thesis. Universidad Icesi, Departamento de Ciencias Naturales, Cali, Colombia.

Schoener, T. W., and G. C. Gorman. 1968. Some niche differences in three Lesser Antillean lizard of the genus *Anolis*. Ecology 49:819-830.

Sifers, S. M., M. L. Yeska, Y. M. Ramos, R. Powell, and J. S. Parmerlee, Jr. 2001. *Anolis* lizards restricted to altered edge habitats in a Hispaniolan cloud forest. Caribbean Journal of Science. 37:55–62.

Smith, J.W., R. Powell, J. S. Parmerlee, Jr., D. D. Smith, and A. Lathrop. 1994. Natural history notes on a population of grass anoles, *Anolis olssoni* (Sauria: Polychrotidae) from the Dominican Republic. Bulletin of the Maryland Herpetological Society. 30:67–75.

Stapley. J., M. Garcia and R. M. Andrews. 2015. Long-term data reveal a population decline of the tropical lizard *Anolis apletophallus*, and a negative affect of *El Nino* years on population growth rate. PLoS ONE 10(2): e0115450.

vanBerkum, F. H. 1986. Evolutionary patterns of the thermal sensitivity of sprint speed in *Anolis* lizards. Evolution. 40:594–604.

vanBerkum, F. H. 1988. Latitudinal patterns of the thermal sensitivity of sprint speed in lizards. The American Naturalist. 132:327–343.

Vitt, L. J., and P. A. Zani. 2005. Ecology and reproduction of *Anolis capito* in rain forest of southeastern Nicaragua. Journal of Herpetology. 39:36–42.

Vitt, L. J., P. A. Zani, and R. D. Durtsche. 1995. Ecology of the lizard *Norops oxylophus* (Polychrotidae) in lowland forest of southeastern Nicaragua. Canadian Journal of Zoology. 73:1918–1927.

Vitt, L. J., S. S. Sartorius, T. C. S. Avila-Pires, and M. C. Espósito. 2001. Life on the leaf litter: The ecology of *Anolis nitens tandai* in the Brazilian Amazon. Copeia. 2001:401–412.

Vitt, L. J., T. C. S. Avila-Pires, P. A. Zani, and M. C. Espósito. 2003a. Life in shade: The ecology of *Anolis trachyderma* (Squamata: Polychrotidae) in Amazonian Ecuador and Brazil, with comparisons to ecologically similar anoles. Copeia. 2002:275–286.

Vitt, L. J., T. C. S. Avila-Pires, M. C. Esp´osito, S. S. Sartorius, and P.A. Zani. 2003b. Sharing Amazonian rain-forest trees: Ecology of *Anolis punctatus* and *Anolis transversalis* (Squamata: Polychrotidae). Journal of Herpetology. 37:276–285.

Wilson, M. A. and A. C. Echternacht. 1987. Geographic variation in the critical thermal minimum of the green anole, A*nolis carolinensis* (sauria: iguanidae), along a latitudinal gradient. Comparative Biochemistry and Physiology. 87A(3): 757-760.

**Table S2:** Description of thermal bioclim variables used in this study.

| **Variable** | **Description** |
| --- | --- |
| bio 1 | Annual Mean Temperature |
| bio 2 | Mean Diurnal Range |
| bio 3 | Isothermality |
| bio 4 | Temperature Seasonality |
| bio 5 | Maximum Temperature of the Warmest Month |
| bio 6 | Minimum Temperature of the Coldest Month |
| bio 7 | Temperature Annual Range |
| bio 8 | Mean Temperature of the Wettest Quarter |
| bio 9 | Mean Temperature of the Driest Quarter |
| bio 10 | Mean Temperature of the Warmest Quarter |
| bio 11 | Mean Temperature of the Coldest Quarter |

**Table S3:** Loadings and eigenvalues from a phylogenetic principal components (PC) analysis on the thermal climate variables from the WorldClim database. Strong loadings shown in bold. Definition of each variable given in **Table S2**.

| **Variable** | **PC 1** | **PC 2** | **PC 3** |
| --- | --- | --- | --- |
| bio 1 | **0.979** | 0.184 | 0.022 |
| bio 2 | -0.289 | 0.387 | 0.132 |
| bio 3 | 0.111 | -0.661 | 0.107 |
| bio 4 | -0.333 | **0.842** | 0.060 |
| bio 5 | **0.810** | 0.555 | 0.058 |
| bio 6 | **0.965** | -0.233 | -0.022 |
| bio 7 | -0.402 | **0.860** | 0.109 |
| bio 8 | **0.912** | 0.372 | 0.038 |
| bio 9 | **0.977** | 0.073 | 0.028 |
| bio 10 | 0.059 | 0.051 | **-0.966** |
| bio 11 | -0.041 | 0.144 | **-0.927** |
| Eigenvalue | 4.71 | 2.59 | 1.82 |
| % Variance Explained | 43.79 | 23.61 | 16.58 |

**Table S4.** Summary of the model fits for the different evolutionary models tested in this study for each body temperature (*T_b_*) in the dataset limited to species with ≥ 10 individuals. The ∆AIC_C_ score refers to the difference between model AIC_C_ and the model with the lowest score. AIC_C_ weight refers to the relative likelihood of the model. BM is a single peak, single rate Brownian motion (BM) model. BMS is a single-peak, two-rate BM model. OU1 is a single-peak, single-rate Ornstein-Uhlenbeck (OU) model. OUM is a two-peak, single-rate OU. OUMV is a two-peak, two-rate OU model. Models with equivalent support (∆AIC_C_ ≤ 4) are shown in bold.

| **BM** | | **BMS** | | **OU1** | | **OUM** | | **OUMV** | |
| --- | --- | --- | --- | --- | --- | --- | --- | --- | --- |
| ∆AIC_C_ | weight | ∆AIC_C_ | weight | ∆AIC_C_ | weight | ∆AIC_C_ | weight | ∆AIC_C_ | weight |
| 18.9 | 0.00 | 21.0 | 0.00 | 7.3 | 0.01 | **0** | **0.60** | **0.9** | **0.39** |

**Table S5:** Results of phylogenetic ANOVAs comparing climatic PC variables and several bioclim variables between mainland and island habitats. For descriptions of PC variables, see **Table S3**.

| **Trait** | ***F*** | ***p*** |
| --- | --- | --- |
| PC 1 | 0.916 | 0.700 |
| PC 2 | 1.816 | 0.576 |
| PC 3 | 0.169 | 0.857 |
| MAT (bio 1) | 0.043 | 0.949 |
| Daily Range (bio 2) | 3.412 | 0.421 |
| Max. Temp. Warmest Month (bio 5) | 0.659 | 0.717 |
| Min. Temp. Coldest Month (bio 6) | 0.309 | 0.819 |
| Temperature Annual Range (bio 7) | 0.057 | 0.919 |

**Table S6.** Summary of the body temperature and environmental (mean annual temperature) data we used in the thermoregulatory analyses. Body temperature and mean annual temperature are given in °C. The landmass type (island or mainland) and geographic region of origin for each species is also given.

| **Species** | **Body Temperature (°C)** | **Mean Annual Temperature (°C)** | **Landmass (Island or Mainland)** | **Geographic Region** |
| --- | --- | --- | --- | --- |
| *Anolis acutus* | 30.92 | 26.2 | Island | Lesser Antilles |
| *Anolis aeneus* | 31 | 25.7 | Island | Lesser Antilles |
| *Anolis aequatorialis* | 22.3 | 16 | Mainland | South America |
| *Anolis agassizi* | 30.6 | 25.3 | Island | Offshore Pacific Island |
| *Anolis aliniger* | 28.83 | 18.9 | Island | Greater Antilles |
| *Anolis allisoni* | 33 | 25.63 | Island | Greater Antilles |
| *Anolis allogus* | 29.2 | 25.2 | Island | Greater Antilles |
| *Anolis alutaceus* | 28.9 | 22 | Island | Greater Antilles |
| *Anolis angusticeps* | 29.2 | 23.2 | Island | Greater Antilles |
| *Anolis apletophallus* | 27.8 | 26.6 | Mainland | Central America |
| *Anolis armouri* | 28.19 | 12 | Island | Greater Antilles |
| *Anolis bahorucoensis* | 24.76 | 26.6 | Island | Greater Antilles |
| *Anolis baleatus* | 28.5 | 25.7 | Island | Greater Antilles |
| *Anolis barbatus* | 27.3 | 23.2 | Island | Greater Antilles |
| *Anolis barbouri* | 27.31 | 20.8 | Island | Greater Antilles |
| *Anolis barkeri* | 24.02 | 17.9 | Mainland | North America |
| *Anolis bartschi* | 26.1 | 24.5 | Island | Greater Antilles |
| *Anolis bimaculatus* | 32.9 | 23.6 | Island | Lesser Antilles |
| *Anolis biscutiger* | 31 | 26.7 | Mainland | Central America |
| *Anolis bonariensis* | 33.4 | 27.2 | Island | Dutch Leeward Islands |
| *Anolis brasilensis* | 31.62 | 19.6 | Mainland | South America |
| *Anolis brevirostris* | 31.33 | 26.5 | Island | Greater Antilles |
| *Anolis capito* | 28.9 | 25.8 | Mainland | Central America |
| *Anolis carolinensis* | 23.7 | 18.8 | Mainland | North America |
| *Anolis carpenteri* | 27.3 | 21.9 | Mainland | Central America |
| *Anolis chlorocyanus* | 30.42 | 24.8 | Island | Greater Antilles |
| *Anolis christophei* | 24.15 | 18.9 | Island | Greater Antilles |
| *Anolis coelestinus* | 27.8 | 18.78 | Island | Greater Antilles |
| *Anolis cooki* | 32.03 | 24 | Island | Greater Antilles |
| *Anolis cristatellus* | 27.38 | 22.61 | Island | Greater Antilles |
| *Anolis cupreus* | 29.4 | 26 | Mainland | Central America |
| *Anolis cuprinus* | 30.7 | 26.8 | Mainland | North America |
| *Anolis cybotes* | 29.66 | 23 | Island | Greater Antilles |
| *Anolis distichus* | 29.26 | 25.3 | Island | Greater Antilles |
| *Anolis dollfusianus* | 28.4 | 27.2 | Mainland | Central America |
| *Anolis gadovii* | 30.5 | 28 | Mainland | North America |
| *Anolis gemmosus* | 21 | 16 | Mainland | South America |
| *Anolis gingivinus* | 30.1 | 26.5 | Island | Lesser Antilles |
| *Anolis grahami* | 31.2 | 24.6 | Island | Greater Antilles |
| *Anolis gundlachi* | 23.75 | 22.9 | Island | Greater Antilles |
| *Anolis heterodermus* | 23.8 | 13.8 | Mainland | South America |
| *Anolis homolechis* | 31.6 | 25.2 | Island | Greater Antilles |
| *Anolis humilis* | 26.2 | 26 | Mainland | Central America |
| *Anolis krugi* | 28.6 | 26 | Island | Greater Antilles |
| *Anolis laeviventris* | 25.96 | 23 | Mainland | Central America |
| *Anolis lemurinus* | 28.8 | 26 | Mainland | Central America |
| *Anolis limifrons* | 26.55 | 26 | Mainland | Central America |
| *Anolis lineatopus* | 28.3 | 24.6 | Island | Greater Antilles |
| *Anolis lionotus* | 26.1 | 26 | Mainland | Central America |
| *Anolis longiceps* | 32.2 | 26.2 | Island | Lesser Antilles |
| *Anolis longitibialis* | 29.21 | 26.1 | Island | Greater Antilles |
| *Anolis loysianus* | 29.02 | 23.2 | Island | Greater Antilles |
| *Anolis lucius* | 29.25 | 25.2 | Island | Greater Antilles |
| *Anolis luteogularis* | 29.4 | 23.2 | Island | Greater Antilles |
| *Anolis marcanoi* | 29.1 | 20.1 | Island | Greater Antilles |
| *Anolis marmoratus* | 29.75 | 22 | Island | Lesser Antilles |
| *Anolis mestrei* | 27.7 | 23.2 | Island | Greater Antilles |
| *Anolis monensis* | 31.29 | 25.5 | Island | Greater Antilles |
| *Anolis nebulosus* | 29.8 | 26.2 | Mainland | North America |
| *Anolis oculatus* | 28.01 | 24.72 | Island | Lesser Antilles |
| *Anolis olssoni* | 32.55 | 25.7 | Island | Greater Antilles |
| *Anolis oxylophus* | 27.77 | 26.6 | Mainland | Central America |
| *Anolis polylepis* | 27.51 | 26.1 | Mainland | Central America |
| *Anolis porcatus* | 32.7 | 23.2 | Island | Greater Antilles |
| *Anolis princeps* | 27.4 | 24.8 | Mainland | South America |
| *Anolis proboscis* | 23.85 | 18.8 | Mainland | South America |
| *Anolis punctatus* | 29.13 | 25.2 | Mainland | South America |
| *Anolis richardi* | 28.4 | 25.7 | Island | Lesser Antilles |
| *Anolis roquet* | 27.22 | 25.6 | Island | Lesser Antilles |
| *Anolis sagrei* | 32.01 | 24.87 | Island | Greater Antilles |
| *Anolis scypheus* | 27.3 | 25.4 | Mainland | South America |
| *Anolis semilineatus* | 31.85 | 22.8 | Island | Greater Antilles |
| *Anolis shrevei* | 29.05 | 11.9 | Island | Greater Antilles |
| *Anolis singularis* | 29.63 | 17.53 | Island | Greater Antilles |
| *Anolis smaragdinus* | 34.2 | 25.8 | Island | Lesser Antilles |
| *Anolis strahmi* | 28.04 | 26 | Island | Greater Antilles |
| *Anolis subocularis* | 31.4 | 28.3 | Mainland | North America |
| *Anolis tandai* | 27.7 | 25.7 | Mainland | South America |
| *Anolis taylori* | 29.1 | 28 | Mainland | North America |
| *Anolis trachyderma* | 27.8 | 25 | Mainland | South America |
| *Anolis transversalis* | 27.6 | 25.2 | Mainland | South America |
| *Anolis tropidolepis* | 19.52 | 24 | Mainland | Central America |
| *Anolis uniformis* | 27.9 | 24.9 | Mainland | Central America |
| *Anolis vermiculatus* | 28.3 | 23.2 | Island | Greater Antilles |
| *Anolis wattsi* | 33.3 | 23.6 | Island | Lesser Antilles |
| *Anolis whitemani* | 27.9 | 24.3 | Island | Greater Antilles |
| *Anolis woodi* | 21.5 | 22.4 | Mainland | Central America |

1. Can be considered synonymous with *A. limifrons*, but treated as separate species in Poe et al. (2017). [↑](#footnote-ref-1)
2. Species values come from unpublished data from R. Huey. Mean values given in Huey et al. (2009) supp. mat. [↑](#footnote-ref-2)
3. Species values come from unpublished data from J. Losos. Mean values given in Hertz et al. (2013) Appendix A. [↑](#footnote-ref-3)
4. Listed as *A. intermedius* in the cited papers. [↑](#footnote-ref-4)
5. Species values come from Campbell (1971). Mean values given in Hertz et al. (2013) Appendix A. [↑](#footnote-ref-5)
6. Species values come from unpublished data from P. Hertz. Mean values given in Hertz et al. (2013) Appendix A. [↑](#footnote-ref-6)
7. Previously known as *A. chocorum*. [↑](#footnote-ref-7)
8. Listed as *A. attenuatus* in Fitch et al. (1976). [↑](#footnote-ref-8)
